# Extremely Elevated International Normalized Ratio in a Patient with Dabigatran Etexilate Use

**DOI:** 10.7759/cureus.4906

**Published:** 2019-06-15

**Authors:** Waqas Memon, Muhammed Aamir, Areeka Memon

**Affiliations:** 1 Internal Medicine, Carle Foundation Hospital, Urbana, USA; 2 Osteopathic Medicine, Edward Via College of Osteopathic Medicine, Blacksburg, USA

**Keywords:** cardiology, dabigatran, supratherapeutic inr, acute kidney injury, idarucizumab, noac

## Abstract

A 76-year-old male with a past medical history (PMH) of hypertension, type 2 diabetes mellitus, chronic kidney disease (CKD) stage three, chronic nonvalvular atrial fibrillation (AF) on anticoagulation, and status post-left-sided nephrectomy in 2000 for acute pyelonephritis presented with multiple episodes of epistaxis and shortness of breath. On exam, the patient was afebrile and saturating 95% on room air. There was crusted blood present in bilateral nares along with a 6 x 3-centimeter area of ecchymosis present on the lateral aspect of the right abdominal wall. Laboratory findings revealed hemoglobin of 6.8 g/dL, hematocrit of 26.5%, bicarbonate of 20.0 mmol/L, blood urea nitrogen (BUN) of 106 mg/dL, creatinine of 3.83 mg/dL, and an INR of >10.0. The patient was initially treated with idarucizumab, which is a monoclonal antibody fragment that binds to dabigatran metabolites and in turn neutralizes dabigatran and the anticoagulant effect of its metabolites. Dabigatran was also discontinued in the setting of elevated creatinine and underlying CKD stage three. After the symptoms resolved, the patient was discharged in a stable condition. Follow-up with the primary care physician (PCP) and cardiology clinic was scheduled for further initiating anticoagulation.

Dabigatran etexilate, when used in patients with renal impairment, has been associated with an increased risk of bleeding in patients. The medication is predominantly excreted by the kidneys (80%) and therefore, renal impairment patients require a reduced dose. There have been multiple reported cases of bleeding related to dabigatran use. However, to the best of our knowledge, this is the first report of an elevated INR of this extreme with the use of dabigatran.

## Introduction

Dabigatran etexilate is the first novel oral anticoagulant (NOAC) approved by the Federal Drug Administration (FDA) for stroke prophylaxis in nonvalvular atrial fibrillation (AF). Renal impairment has been associated with an increased risk of bleeding in patients who are on dabigatran etexilate. There have been multiple reported cases of bleeding related to dabigatran use [1,2]. Patients who are started on dabigatran etexilate, usually do not have renal function assessed and this is a cause of morbidity, especially worsening outcomes related to bleeding [3]. Currently, guidelines do not indicate routine prothrombin time/international normalized ratio (PT/INR) [3]. To the best of our knowledge, this is the first report of an extremely elevated INR with the use of dabigatran.

## Case presentation

A 76-year-old male with a past medical history of hypertension, diabetes mellitus type two, chronic kidney disease (CKD) stage three, chronic nonvalvular atrial fibrillation on anticoagulation with a CHA2DS2-VASc Score = 3, and status post-left-sided nephrectomy in 2000 for acute pyelonephritis presented with multiple episodes of profuse epistaxis and shortness of breath with daily activity that had been occurring for four hours prior to admission. Home medications included aspirin 81 mg, furosemide 20 mg, lisinopril 20 mg, and simvastatin. On exam, the patient had a temperature of 36.6 C, blood pressure of 103/54 mm Hg, heart rate of 69 beats per minute, and saturating 95% on room air. There was crusted blood present in bilateral nares along with a 6 x 3-centimeter area of ecchymosis present on the lateral aspect of the right abdominal wall. Laboratory findings revealed hemoglobin of 6.8 g/dL, hematocrit of 26.5%, bicarbonate of 20.0 mmol/L, blood urea nitrogen (BUN) of 106 mg/dL, creatinine of 3.83 mg/dL, and an INR of >10.0 ratio. The patient was initially treated with one dose of 5 mg idarucizumab, and dabigatran was discontinued in the setting of elevated creatinine and underlying CKD stage three. The patient was given one unit of packed red blood cells (PRBC) and the hemoglobin increased to 8.7 g/dL. Fecal occult blood was positive and gastroenterology was consulted. An upper endoscopy was done and it revealed gastritis but no sites of bleeding. After the symptoms resolved, the patient was discharged in a stable condition. He was started on pantoprazole and scheduled for a primary care physician (PCP) and cardiology clinic follow-up for further initiation of anticoagulation.

## Discussion

Dabigatran etexilate was the first novel oral anticoagulant (NOAC) that was approved by the FDA for stroke prophylaxis in nonvalvular atrial fibrillation. Renal impairment has been associated with an increased risk of bleeding in patients who are on dabigatran etexilate [3]. The medication is predominantly excreted by the kidneys (80%). Therefore, renal impairment patients require a reduced dose. The FDA recommends patients with a creatinine clearance of 15-30 mL/min, to use a reduced dosage of 75 mg twice daily. The medication is contraindicated in patients with a creatinine clearance less than 15 mL/min in acute renal failure or end-stage renal disease (ESRD). However, no outcome data exists for newer anticoagulants with a creatinine clearance less than 30 mL/min, and use is advised against in this patient population [3].

Patients who are started on dabigatran etexilate usually do not have renal function assessed and this is a cause of morbidity [4,5]. Once the medication is started, it is difficult to assess the anticoagulant effect [1,6]. In the hospital setting, there is no available laboratory study to confirm dabigatran etexilate-induced coagulopathy [2].

Vidal et al. reported a case in which a 73-year-old female with deep vein thrombosis (DVT) on dabigatran developed rectal bleeding during the course of hospitalization. The patient had a rise in INR from 1.4 to 2.3 and it was successfully reversed with idarucizumab [2]. The 'Reversal Effects of Idarucizumab on Active Dabigatran' (RE-VERSE AD) was a prospective cohort study that showed that idarucizumab administration reversed anticoagulation as evidenced by the normalization of the dilute thrombin time and ecarin clotting time within minutes among subjects suffering a serious hemorrhage [7].

INR does not directly correlate with the activity of dabigatran; however, after initial management with idarucizumab, the PT/INR decreased from >10 to 1.7 [2]. Our case is an exception in regards to very high supratherapeutic INR of > 10 likely secondary to dabigatran use, which has never been reported before. Our patient required only 5 g of idarucizumab for the complete reversal of INR, as well as clinical recovery from epistaxis.

## Conclusions

Bleeding complications with dabigatran have been reported by multiple case reports. To the best of our knowledge, this is the first reported case of extremely elevated INR with the use of dabigatran. Currently, guidelines do not indicate routine PT/INR follow-up in patients taking dabigatran. When determining the need to start a patient on dabigatran, it is crucial that patients have their renal function checked prior to starting therapy and that the medication is dosed based on creatinine clearance. In the acute setting, there is no reliable laboratory study to monitor the anticoagulant effect; hence, it is beneficial to check PT and activated partial thromboplastin time (aPTT). In patients with decreased renal function, warfarin should be regarded as an option to decrease the risk of bleeding. Forthcoming studies are warranted to help discover a safe dose of dabigatran in patients with renal impairment and a way of monitoring the activity of dabigatran.
